# Medical Opinion and Movement

**Published:** 1906-12-08

**Authors:** 


### Medical Opinion and Movement.

It is with the deepest regret that we have to record the sudden death of Dr. George Bagot Ferguson, of Cheltenham. Dr. Ferguson was much esteemed in Cheltenham and Gloucestershire, and has for many years taken a prominent part in the doings of the Gloucestershire Division of the British Medical Association; but he was known and respected by a much wider circle of medical men owing to the able and genial manner in which he carried out his duties as President at the annual meeting of the Association held at Cheltenham in 1901. While operating at the Cheltenham Infirmary on Tuesday afternoon, November 27, upon a case of strangulated hernia, requiring resection of the gut, he was suddenly seized with an attack of angina pectoris, and fell back dead. His colleague, Mr. Howell, was sent for, and he completed the operation. We understand that Dr. Ferguson has been suffering from attacks of angina recently, and from other symptoms of heart trouble. A resolution expressing the warmest sympathy with Mrs. Ferguson and her family was unanimously adopted at the general meeting of the British Medical Association on the following day.

The General Medical Council commenced their winter session on Monday, November 27. In the opening address of the President he was able to show that through representations made by the Executive Committee to the Privy Council they had been instrumental in frustrating the attempts of two public bodies to obtain powers which were deemed detrimental to the medical profession and dangerous to the public. The National Association of Medical Herbalists of Great Britain, Limited, petitioned for a charter of incorporation. The grant of such a charter would be tantamount to the legalisation of quackery. In accordance, however, with the advice of the Executive Committee, drawn up in a memorandum to the Lord President, this petition has been refused. A like fate has been meted out to the petition for a charter by the British Optical Association, and it is understood that the Sight Testing Opticians' Bill, introduced into the House of Lords with a similar purpose, has been dropped. Among other subjects of interest, the President referred to his visit to Canada, and stated that, as a result of his interviews with Ministers of the Crown and other influential persons, he was convinced that further medical reciprocity between the Dominion and the Mother Country should be encouraged, and that "nothing but good can result from a closer connection and a freer interchange between the professional schools there and here."

The motion of Sir Thomas Myles to instruct the Examination Committee to report upon the advisability of dispensing with compulsory attendance upon systematic lectures in the medical schools so as to set free more time for scientific study raises the interesting question of the value of lectures. In spite of the many excellent text-books now at the command of the student of any subject, there is no doubt that a good lecture delivered by one who has not only a thorough knowledge of the subject in hand, but, above all, has the ability to lecture, is an excellent supplement to the private reading and practical work of the student. The lecturer is able to illuminate the subject by his own personality and by his experience, to emphasise and underline the essential features, and make them stand out in relief in a manner which it is impossible to do in a text-book. The difficulty arises in the scarcity of good lecturers. Whether it be due to a lack of natural ability or to a neglect of the art of lecturing, it is an undoubted fact that but a small proportion of the total number of medical men, whose duty it is to deliver lectures at the different London medical schools, have the power to engage the attention and enlist the interest of the medical student. This is probably largely due to the unfortunate custom which has arisen in the medical schools of appointing members of the staffs of the corresponding hospitals to lectureships more or less in rotation, and without any consideration of their abilities to lecture. When the students are fortunate enough to secure a really efficient lecturer they are never slow to show their appreciation of his services by regular attendance and earnest attention.

The long outstanding dispute between the Coventry Provident Dispensary has entered upon a new and somewhat acute phase. In consequence of the pressure put upon the management of the dispensary by the profession to take steps to abolish the abuses in connection with it, the committee have adopted a measure of retort which can only be regarded as wholly unworthy of any public body. Dr. Snell, who is the Honorary Secretary of the Coventry Division of the British Medical Association, is also the medical officer of the city of Coventry. The committee have accordingly addressed a letter to the Mayor, Aldermen, and Councillors of the city of Coventry, in which they draw their attention to the fact that the dispensary is placed upon the "black list" of the *British Medical Journal*, and that Dr. Snell, acting as Honorary Secretary of the local division of the Association, is largely responsible for this action. They ask whether such action is in accordance with their wishes and the right discharge of Dr. Snell's public duties. The letter was referred to the Sanitary Committee, and they resolved—

> That, insomuch as Dr. Snell, in acting as Honorary Secretary, is discharging an honorary office, and acting purely within his private capacity, the committee consider that the matter does not concern them.

The Council referred this to the Sanitary Committee on two occasions. It is satisfactory to see that the Sanitary Committee of the Council have shown a proper readiness to support their medical officer, and it is to be hoped that sentiments of fairness and justice may eventually prevail with the Council. It is an obvious intrusion upon the private liberty of the individual to make him accountable to his employers, be they a public or private body, for any work he may choose to take up in his leisure hours.

